# Depressive symptoms among heterosexual and sexual minority college students: network analyses

**DOI:** 10.1192/j.eurpsy.2025.1307

**Published:** 2025-08-26

**Authors:** E. B. Camargo Júnior, G. G. Silva, M. N. D. F. Fernandes, E. C. D. S. Gherardi-Donato

**Affiliations:** 1 University of Rio Verde, Rio Verde; 2 Federal University of Maranhão, Imperatriz; 3 Ribeirão Preto College of Nursing, Ribeirão Preto, University of São Paulo, Ribeirão Preto, Brazil

## Abstract

**Introduction:**

Depression is one of the most prevalent mental disorders worldwide, and network analyses in psychopathology allow for an improved understanding of its development, maintenance, and treatment. Evidence suggests that sexual minority individuals are more vulnerable to depression.

**Objectives:**

The objective of this study was to analyze the correlations between depressive symptoms in heterosexual and sexual minority students using network analysis.

**Methods:**

This cross-sectional study was conducted with 1,271 university students to assess depressive symptoms using the Patient Health Questionnaire-9 (PHQ-9). Network analyses were performed to identify influential symptoms and their correlations.

**Results:**

The results showed that sexual minority students had significantly higher scores for all symptoms of depression than heterosexual students did. Guilt and suicidal ideation had the strongest association with the symptom network of heterosexual students. Among sexual minority students, the strongest associations were between lack of energy and depressed moods. The most influential symptom in the heterosexual student network was suicidal ideation, whereas in the sexual minority student network, guilt was the most influential symptom.

**Conclusions:**

These results demonstrate that in network analyses, different depressive symptoms play distinct central roles between heterosexual and sexual minority groups. These differences indicate the need for interventions that are adapted to the specificities of each group to promote mental health.

**Disclosure of Interest:**

None Declared

